# Optopharmacology reveals a differential contribution of *native* GABA_A_ receptors to dendritic and somatic inhibition using azogabazine

**DOI:** 10.1016/j.neuropharm.2020.108135

**Published:** 2020-10-01

**Authors:** Martin Mortensen, Rosemary Huckvale, Arun P. Pandurangan, James R. Baker, Trevor G. Smart

**Affiliations:** a Department of Neuroscience, Physiology and Pharmacology, University College London, Gower Street, London, WC1E 6BT, UK; b The Institute of Cancer Research, 123 Old Brompton Road, London, SW7 3RP, UK; c MRC Laboratory of Molecular Biology, Francis Crick Avenue, Cambridge Biomedical Campus, Cambridge, CB2 0QH, UK; d Department of Chemistry, University College London, 20 Gordon Street, London, WC1H 0AJ, UK

**Keywords:** GABA_A_ receptor, Synaptic inhibition, Azobenzene, Optopharmacology, Photoisomerisation, Gabazine, Azogabazine, GABA, Homology modelling, Computational ligand docking, Cerebellar granule neurons

## Abstract

γ-aminobutyric acid type-A receptors (GABA_A_Rs) are inhibitory ligand-gated ion channels in the brain that are crucial for controlling neuronal excitation. To explore their physiological roles in cellular and neural network activity, it is important to understand why specific GABA_A_R isoforms are distributed not only to various brain regions and cell types, but also to specific areas of the membrane in individual neurons. To address this aim we have developed a novel photosensitive compound, azogabazine, that targets and reversibly inhibits GABA_A_Rs. The receptor selectivity of the compound is based on the competitive antagonist, gabazine, and photosensitivity is conferred by a photoisomerisable azobenzene group. Azogabazine can exist in either *cis* or *trans* conformations that are controlled by UV and blue light respectively, to affect receptor inhibition. We report that the *trans*-isomer preferentially binds and inhibits GABA_A_R function, whilst promotion of the *cis*-isomer caused unbinding of azogabazine from GABA_A_Rs. Using cultured cerebellar granule cells, azogabazine in conjunction with UV light applied to defined membrane domains, revealed higher densities of GABA_A_Rs at somatic inhibitory synapses compared to those populating proximal dendritic zones, even though the latter displayed a higher number of synapses per unit area of membrane. Azogabazine also revealed more pronounced GABA-mediated inhibition of action potential firing in proximal dendrites compared to the soma. Overall, azogabazine is a valuable addition to the photochemical toolkit that can be used to interrogate GABA_A_R function and inhibition.

## Highlights

• Azogabazine is a new photoactivated reversible antagonist at GABA_A_ receptors.
• Inhibition can be reversibly controlled by blue and UV light.
• Azogabazine binds at the GABA_A_ receptor's orthosteric binding site.
• Photo-inhibition revealed differential GABA_A_R numbers at somatic and dendritic neuronal zones.
• Azogabazine is a novel tool for investigating GABA_A_ receptor function and distribution.

## 1 Introduction

γ-aminobutyric acid (GABA) is the main inhibitory neurotransmitter in the central nervous system (CNS) causing rapid inhibition of neurons by activating pentameric GABA_A_Rs (Smart and Paoletti, 2012). The physiological and pharmacological characteristics of these receptors are determined by their subunit composition which can be selected from 19 different isoforms (α1-6, β1-3, γ1-3, δ, *ε*, θ, π, and ρ1-3; Sieghart and Sperk, 2002). Classical synaptic-type GABA_A_Rs are composed of αβγ subunits (2:2:1 ratio) where α and γ subunits are instrumental in locating the receptors to scaffold proteins such as gephyrin and GARHLs, in the postsynaptic density (Luscher et al., 2011; Yamasaki et al., 2017; Jacob et al., 2008). Extrasynaptic receptors are uniquely composed of αβδ and αβ receptors (Mortensen and Smart, 2006; Brickley and Mody, 2012; Brickley et al., 1999; Glykys et al., 2008), and also include αβγ GABA_A_Rs, since these translocate laterally between synaptic and extrasynaptic zones and are therefore not exclusively located at inhibitory synapses (Thomas et al., 2005; Bogdanov et al., 2006).

Dysfunctional GABA_A_Rs are also implicated in a swathe of neurological disorders including anxiety and depression, epilepsy, schizophrenia and sleep disorders (Möhler, 2012; Rudolph and Möhler, 2014; Kang and Macdonald, 2009; Hines et al., 2012; Ramamoorthi and Lin, 2011). As such these receptors are targeted by important drug classes including the benzodiazepines, general anaesthetics, neuroactive steroids and barbiturates (Korpi et al., 2002; Sieghart and Savić, 2018). To probe GABA_A_R function in the nervous system, and to exploit new designs of drug molecules that can be rapidly activated or inactivated, we and others have recently developed new research tools based on photochemically-active compounds that target GABA_A_Rs (Mortensen et al., 2014; Iqbal et al., 2011; Lin et al., 2014, 2015; Yue et al., 2012). To develop a compound that can reversibly and repeatedly inhibit targeted GABA_A_Rs we have modified the antagonist gabazine to enable light-activated *cis*:*trans* conformational isomerization (Huckvale et al., 2016). This new photochemical molecule, called azogabazine (AGZ), has been used to explore the mechanism of antagonist-based isomerization, and how this may be used to dissect synaptic inhibition by targeting the proximal-dendritic and somatic regions of neurons using membrane delimited-photoisomerisation. This revealed differential contributions of GABA_A_Rs located in discrete cell membrane sub-domains to inhibition of cerebellar granule neurons.

## 2 Methods

### 2.1 Organic chemistry, ^1^H NMR and drug handling

The synthesis of azogabazine, and structure determination using proton nuclear magnetic resonance (^1^H NMR) was described previously (Huckvale et al., 2016). In brief, 4-aminophenylboronic acid pinacol ester was synthesized from iodoaniline and underwent a condensation reaction with nitrosobenzene to form the azobenzene building block, which was isolated after conversion to the tri-fluoroborate salt and recrystalisation from acetone. Palladium-catalysed cross-coupling reaction with 3-amino-6-chloropyridazine, followed by N-alkylation of the pyridazine moiety with allyl 4-bromobutyrate and subsequent ester hydrolysis yielded azogabazine.

For most of the study azogabazine was kept in darkness to ensure a stock of near-100% *trans*-isomer. Unless stated otherwise, experiments were performed in near-darkness (under red light) when making azogabazine stocks (1 mM stock solution in DMSO), and when pre-exposing stock solutions to UV or blue light using wavelength specific LEDs. Pre-exposure of azogabazine (stock) to UV or blue light was undertaken by positioning a UV (365 nm) or blue (470 nm) OptoLED (Cairn Research Ltd, Faversham, UK) 2–3 mm above a 500 μl droplet on parafilm and exposing for 2 min at maximum light intensity to ensure attainment of equilibrium between *cis* and *trans* isomers. The UV LED was operated at 1.2 A to achieve a light intensity output at the surface of the droplet of 5.3 mW. By comparison, the Blue LED was run at 0.9 A for a light intensity output of 8.1 mW at the surface of the droplet.

### 2.2 HEK293 cell culture and primary culture of cerebellar granule cells

Human embryonic kidney cells (HEK293) were maintained in Dulbecco's modified Eagle's medium (DMEM) supplemented with 10% v/v fetal bovine serum, 2 mM glutamine and 100u/ml penicillin-G and 100 μg/ml streptomycin at 37 °C in humidified air with 5% CO_2_. Before DNA transfection, HEK293 cells were seeded onto poly-l-lysine coated 22 mm glass coverslips.

Dissociated cerebellar cultures were prepared from cerebellar tissue taken from postnatal day 4 (P4) Sprague Dawley rats. Tissue blocks were incubated in trypsin for 10 min (0.1% w/v), washed in Hanks Balanced Salt Solution (HBSS), and then triturated in DNase (0.05% w/v in 12 mM MgSO_4_). Cells were plated on poly-l-ornithine coated 22 mm glass coverslips in Basal Medium Eagle (BME) supplemented with 0.5% (w/v) glucose, 5 mg/l insulin, 5 mg/l transferrin, 5 mg/l selenium, 20 u/ml penicillin-G and 20 μg/ml streptomycin, 0.2 mM glutamine, 1.2 mM NaCl, and 5% (v/v) fetal calf serum. The neuronal culture was then allowed to develop for 7–10 days at 37 °C in humidified air with 5% CO_2_ before used for experiments.

### 2.3 GABA_A_R constructs and transfecting HEK293 cells

Point mutations were introduced into murine GABA_A_R subunit cDNA in pRK5 by reverse PCR using standard techniques and confirmed by full-length DNA sequence analysis. HEK293 cells were transfected with cDNAs encoding for GABA_A_R α1 (wild-type and mutants), β2 (wild-type and mutant), γ2L (wild-type), and eGFP in a ratio of 1:1:1:1, using a standard calcium phosphate method with 340 mM CaCl_2_ and HBSS (50 mM HEPES, 280 mM NaCl and 2.8 mM Na_2_HPO_4_, pH 7.2) to form a precipitate. Following transfection (16–48 h), expressing cells were identified by GFP fluorescence prior to electrophysiology.

### 2.4 Electrophysiology and photo-isomerization

Cover slips with transfected cells or cultured neurons were placed in a recording chamber on a Nikon Eclipse FN1 microscope incorporating a bespoke optical-illumination system (Cairn Research, Faversham, UK). All azogabazine solutions were shielded from light and the room was maintained in near-darkness with additional dark screening around the electrophysiology rig.

Cells were continuously perfused with Krebs solution containing (mM): 140 NaCl, 4.7 KCl, 1.2 MgCl_2_, 2.52 CaCl_2_, 11 Glucose and 5 HEPES (pH 7.4). In voltage-clamp experiments, patch pipettes were filled with an intracellular solution containing (mM): 140 CsCl, 2 NaCl, 2 MgCl_2_, 5 EGTA, 10 HEPES, 0.5 CaCl_2_, 2 Na-ATP and 0.5 Na-GTP (pH 7.2). For current-clamp experiments on cerebellar granule cells, patch pipettes were filled with a solution containing (mM): 137 K-gluconate, 3 KCl, 2 MgCl_2_, 5 EGTA, 10 HEPES, 0.5 CaCl_2_, 2 Na-ATP and 0.5 Na-GTP (pH 7.3). For HEK293 cell recordings we used thin-walled, filamented borosilicate glass capillaries (TW150F-4; WPI, USA), with resistances 3–4 MΩ. For cerebellar granule cells thick-walled, filamented borosilicate glass capillaries (1B150F-4; WPI) were used with resistances of 6–8 MΩ.

To isolate GABA-mediated sIPSCs in cerebellar granule cell experiments, the Krebs solution contained CNQX (10 μM) and AP-5 (20 μM) to inhibit excitatory synaptic currents. Drugs were applied to cells using a U-tube application system (Mortensen and Smart, 2007).

Cells were voltage- or current-clamped at −60 mV with an Axopatch 200B amplifier (Molecular Devices, USA). Whole-cell currents were filtered at 5 kHz (−36 dB), digitized at 50 kHz via a Digidata 1322A (Molecular Devices), and recorded to disk (Dell Optiplex 990) using Clampex 10.2. In voltage-clamp experiments, cells were series resistance compensated at 60–70%, and monitored throughout each experiment. Deviations of more than 20% resulted in the data being excluded from further analysis.

To initiate photo-isomerization of azogabazine, optical light paths were used to convey UV laser (Obis [375 nm], Coherent Inc., Santa Clara, USA) or blue LED light (OptoLED [470 nm], Cairn Research Ltd, Faversham, U.K.) through a variable diaphragm (for setting the illuminated spot-diameter), and finally through a water-immersion objective (Nikon, NIR Apo 40×/0.8w) to the cells. The diaphragm was kept fully open for whole cell light exposure experiments and reduced to generate small spots (~8–12 μm) when exposing soma or proximal dendritic areas. The illuminated spot size and position could be adjusted during experiments. Whereas the blue LED could be operated at high power (8.1 mW) during experiments, the UV laser output was empirically adjusted to 2 mW (4% of maximum operating power) to avoid cell damage, but still sufficient to adequately azogabazine isomerization. To adjust the size and position of the illuminated spot, a 440 nm white LED pilot light and a 515 nm long-pass filter were used before switching to UV or blue light.

### 2.5 Analysis of electrophysiology data

The potency of azogabazine was evaluated by constructing inhibition-concentration relationships from peak GABA currents and fitting the data using the following equation:

I/I_max_ = 1 – [1/1 + (IC_50_/B)^n^]

where the IC_50_ is the antagonist concentration (B) causing half-maximal inhibition of an EC_50_ GABA induced response, n is the slope factor. IC_50_ values obtained from individual experiments were converted to pIC_50_ values (-Log IC_50_) before an average pIC_50_ value ± SE was calculated. This mean pIC_50_ values were then converted to a mean IC_50_ value.

IPSCs from cerebellar granule cells were detected *post-hoc* using WinEDR 3.5.6, and analysed for frequency, amplitude, rise time and weighted decay tau using WinWCP 4.9.6. Action potentials from cerebellar granule cells were recorded under current-clamp and were similarly detected with WinEDR 3.5.6. Data were subjected to statistical analyses (ANOVA, paired and unpaired Student t-tests).

Peak-scaled non-stationary variance analysis was used with somatic and dendritic synaptic GABA currents to deduce single channel current and average synaptic receptor numbers open at the peak of the IPSC. GABA IPSCs were selected according to clean rise and decay phases. These IPSCs were imported into WinWCP v5.2.3 (John Dempster, University of Strathclyde), and their peaks scaled with currents also aligned according to their negative rising phases. The decay phases of individual IPSCs were subtracted from the mean sIPSC decay to generate the IPSC variance which was plotted against the corresponding mean current amplitude according to:

σ^2^ = [i.I_m –_ (I_m_^2^/N)] + Var_b_

Where σ^2^ is the current decay variance, i is the single channel current, I_m_ is the mean synaptic current and N is the average number of synaptic receptors activated during an IPSC. Var_b_ represents the baseline current variance. This equation was used to generate parabolic curve fits to the current variance – mean current relationships and then used to estimate i and N for synaptic GABA_A_Rs by non-linear least squares regression analysis.

### 2.6 Structural homology modelling and computational docking

A homology model for the murine GABA_A_R isoform, α1β2γ2L, was built using the cryo-EM structure of human α1β3γ2L as a template (pdb: 6I53; Laverty et al., 2019). Amino acid sequences for murine GABA_A_R α1, β2, γ2L subunits were aligned to the human α1, β3, γ2L subunit sequences using ClustalW with manual adjustment. Modeller (ver. 9.19; Sali and Blundell, 1993) was used to generate 50 different models of murine α1β2γ2L using 6I53 as a structural template. The model quality estimation webtool for membrane proteins, QMEANBrane (http://swissmodel.expasy.org/qmean/), was used to rank all 50 models to obtain the best model, which was subsequently processed in SCRWL4 (Krivov et al., 2009) to optimise side-chain configurations. Further model optimization was performed with MOLProbity (http://molprobity.biochem.duke.edu/). The α1β2γ2L GABA_A_R homology model was then structure minimized in Chimera, before a final run through MOLProbity where protein geometry scores showed successful continued model optimization leading to our final homology model. All structural images were visualized and rendered using the molecular graphics system, PyMOL (Schrödinger) and Chimera (Pettersen et al., 2004).

Using Hermes version 1.104.2 interface and GOLD version 5.7.2 (Jones et al., 1997), we docked azogabazine into the orthosteric GABA binding site of the α1β2γ2L GABA_A_R model. Ten residues found within a sphere of 15 Å radius from the α carbon of β2^Y157^ were kept fully flexible during docking (Mortensen et al., 2014). We performed two diverse docking runs. Firstly, 50 different docking solutions were generated for each of the following scoring functions: CHEMPLP (Piecewise Linear Potential), GoldScore (GS), ChemScore (CS) and Astex Statistical Potential (ASP), available from GOLD by restricting rotation within the azogabazine molecule to maintain planarity amongst the aromatic groups. Secondly, a run was designed to test the possibility of azogabazine adopting the *cis* conformation by allowing full rotational degrees of freedom. This latter run generated 100 different docking solutions using the CHEMPLP scoring function. Hydrogen bonding and cation-π interactions were also predicted.

## 3 Results

### 3.1 Competitive GABA_A_R antagonist incorporating a photo-active azobenzene group

**Fig. 1:**
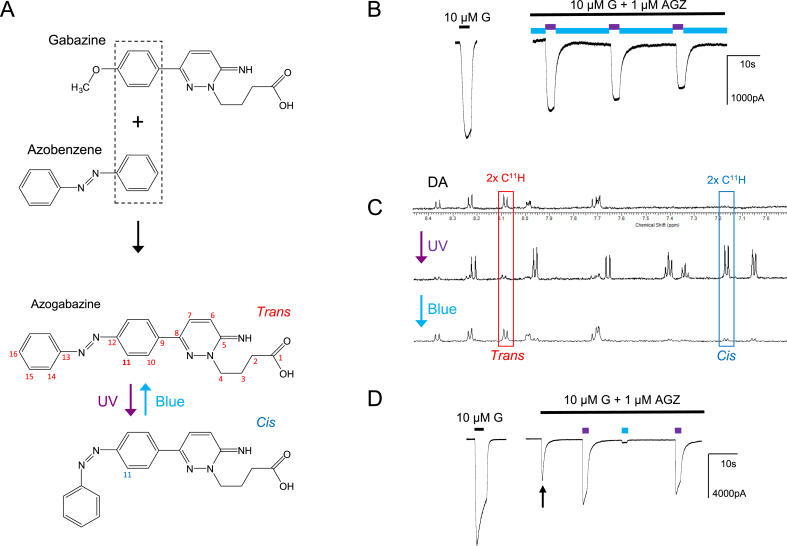
Isomerization of azogabazine and GABA_A_R inhibition. (**A**), Top panel, structures for gabazine and azobenzene, highlighting a shared benzene ring (box) in the fusion-structure of azogabazine. Lower panel, *cis* and *trans* conformations of azogabazine induced by UV and blue light. Carbon atom numbering for ^1^H NMR analysis is shown (red) with C^11^ in bold. (**B**), GABA_A_R currents activated by 10 μM GABA (G) for α1β2γ2L GABA_A_Rs expressed in HEK293 cells (left, control) and after co-application with 1 μM azogabazine (AGZ, right) during sequential exposure to either blue light (blue bar, 470 nm) or UV light (purple bar, 365 nm). (**C**), Top line, ^1^H NMR spectra for a dark adapted (DA) azogabazine solution; Middle line, same sample after UV (365 nm) exposure for 60s; Lower line, and following exposure to blue (470 nm) light for 60 s. Note changes in signal intensity and chemical shift (ppm) for protons at C^11^ on isomerization from *trans*- (red) to *cis* (blue)-azogabazine. (**D**), GABA (10 μM)-activated currents for α1β2γ2L GABA_A_Rs briefly exposed to either dark, UV or blue light. Initial co-application of GABA and AGZ reveals the onset of block (arrow). All recordings were performed at −60 mV. Representative examples are shown from n = 3 NMR experiments and n = 7 voltage clamp experiments.

Previously, we designed and created a series of photoactive chemical tools based on the potent competitive GABA_A_R antagonist gabazine as the functional moiety, which also incorporated a benzophenone group. This latter group was specifically designed to enable covalent binding to the orthosteric GABA binding site and thus cause irreversible receptor antagonism (Iqbal et al., 2011; Mortensen et al., 2014). Although a valuable reagent, it did not permit reversible antagonism. To diversify the range of GABA photochemical tools, and incorporate reversibility, we have created a new molecule where gabazine is coupled to an azobenzene group. In creating this molecule, the fusion of gabazine and azobenzene involved sharing a common phenyl ring leading to the photoisomerisable gabazine analogue, azogabazine (Fig. 1A; Huckvale et al., 2016). The significance of using the azo linkage is that it flips from *cis* to *trans* isomers on light exposure fundamentally altering the conformation of the molecule and thus its pharmacological properties (Beharry and Woolley, 2011; Broichhagen and Trauner, 2014). On this basis we predicted the azobenzene group would enable gabazine to become a reversible photo-activatable GABA_A_R antagonist that could be functionally regulated simply by switching between two different wavelengths of light. Accordingly, exposure to blue light (wavelength (*λ*) 470 nm) would cause azogabazine to adopt a predominant *trans* (approximately planar) *E* form conformation (Zhu and Zhou, 2018), whereas UV light (*λ* 365 nm) would favour the formation of the *cis*-isomer which adopts a non-planar, twisted conformation *Z* form (Fig. 1A). This interconversion is chemically readily reproducible and should enable rapid reversible light-driven receptor antagonism.

The functional properties of azogabazine were assessed initially by expressing a classical synaptic-type recombinant GABA_A_R composed of α1β2γ2L in HEK293 cells and using patch clamp electrophysiology. Azogabazine had previously proved to be an effective antagonist in normal ambient daylight (pIC_50_: 7.65 ± 0.14 (IC_50_: 22.6 nM); Huckvale et al., 2016), and when duty-cycling exposure between blue and UV light, the GABA current rapidly flipped between inhibition and relief. Brief UV light exposure enabled the GABA current to recover temporarily to 64 ± 9% (n = 8) of the control GABA current amplitude (Fig. 1B).

### 3.2 Azogabazine binding to the orthosteric GABA binding site

**Fig. 2:**
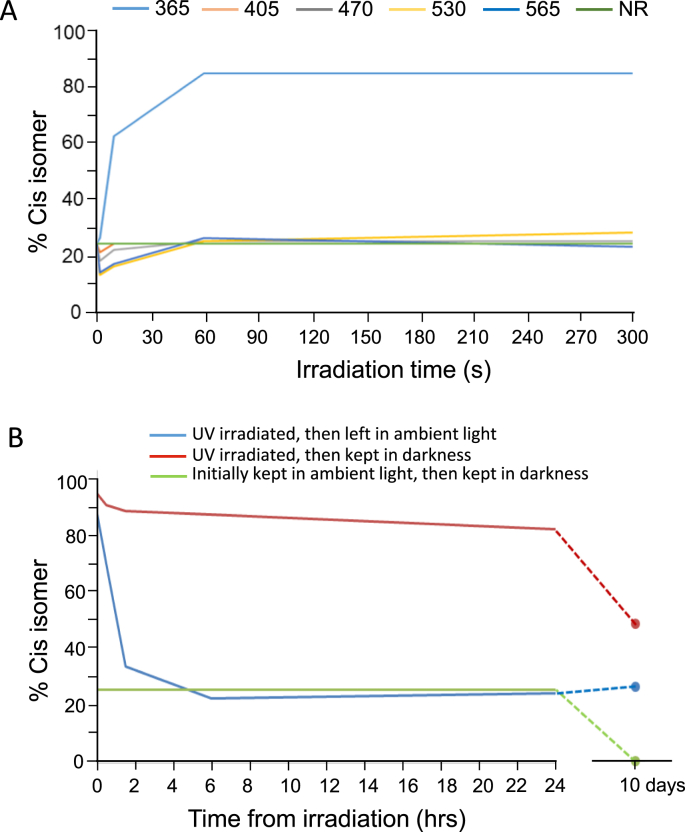
Azogabazine isomerization and isomer stability. (**A**), Stability plot for the *cis*-isomer for non-dark adapted azogabazine samples exposed to different wavelengths of light: *λ* 365, 405, 470, 530, 565 nm, and near-infrared (NR), prior to ^1^H NMR. Note only UV light (365 nm, blue line) resulted in efficient *trans*-to-*cis* isomerization. A long (60 s) period of UV-exposure was needed to reach a maximal equilibrium conversion ratio of ~85 : 15% for *cis: trans* isomers. As the starting samples had been exposed to ambient daylight the blue light (470 nm, grey line) did not change the *cis:trans* ratio (remaining at ~25% *cis*: ~75% *trans*). (**B**), The stability of a UV exposed and established *cis: trans* ratio is dependent on subsequent storage conditions. If the UV-exposed sample is subsequently kept in ambient light (blue line), the *cis:trans* ratio changes over ~ 2–4 h towards ~25% *cis*: 75% *trans* remaining stable over 10 days (dashed blue line). By contrast, the UV-exposed sample remained stable (~85% *cis*: 15% *trans*) over 24 h if subsequently kept in darkness (red line). However, over 10 days, conversion to a predominant *trans*-form was evident (dashed red line). Finally, when a sample which had been kept in ambient light (~25% *cis*: 75% *trans*) was then placed and stored in darkness, it remained stable over 24 h (green line) but slowly converted again to the *trans* form over 10 days (dashed green line).

By fusing the azobenzene group to gabazine, the light-induced isomerization between *cis* and *trans* isomers clearly affected the functionality of the molecule, one being an active isomer and the other inactive as a GABA antagonist. To physically assess the photo-conversion efficiency between the *cis* and *trans* isomers we determined the proton (^1^H)-NMR spectra for azogabazine (10 μM) in deuterated DMSO under different light conditions. Initially, azogabazine was retained in complete darkness for >14 days and then stored under similar dark conditions. This drove the *cis:trans* equilibrium almost completely towards the more thermodynamically stable *trans* isomer (Fig. 1C). The ^1^H NMR spectra revealed a diagnostic proton on C-11 (Fig. 1A,C) that when deshielded in the *trans* isomer gave rise to a doublet at approximately 8.08 ppm (red box, Fig. 1C). By exposing azogabazine to UV light at 365 nm for 60 s (an exposure-time required for complete isomerization; Fig. 2A), the previously dominant proton coupling at C-11 was significantly reduced and a new doublet appeared at ~7.17 ppm, indicative of the *cis* isomer (blue box, Fig. 1C). By integrating the ^1^H NMR spectra, long-term darkness produced ~100% of *trans-*azogabazine, whereas the UV (365 nm) exposure produced an equilibrium of ~85% *cis* to 15% *trans* isomers. Exposing the azogabazine solution to blue light at 470 nm for 60 s resulted in a new photostationary state of approximately 75% *trans*-azogabazine to 25% in the *cis* conformation (Fig. 1C). Thus, switching the wavelength of light produced substantive photoisomerisation of azogabazine.

The consequences of switching between *cis* and *trans* forms of azogabazine were ascertained from a background of ambient daylight and using a duty-cycle light protocol that sequentially exposed cells to UV or blue light in the presence of 1 μM azogabazine (not-dark adapted, and therefore consisting of ~75% *trans*-: ~25% *cis-*azogabazine) and 10 μM GABA, both applied to HEK293 cells expressing α1β2γ2L receptors. Control GABA currents were first established and then GABA and azogabazine were co-applied, initially in ambient daylight. The declining GABA current most likely reflected a slow binding rate for azogabazine compared to GABA, which nevertheless ultimately led to a near complete block of GABA current by azogabazine (arrow, Fig. 1D). Following exposure to UV light (causing the *trans*:*cis* ratio of 75 : 25% to shift mostly towards the *cis* form - ratio 15 : 85%), the GABA current recovered to 66 ± 9% (n = 8) of control GABA responses. This indicated that the *trans* isomer was the most effective antagonist binding to the GABA_A_R, and that it would unbind when isomerized to *cis*-azogabazine. By switching to blue light (converting azogabazine to the *trans* form) inhibition was mostly maintained with only a small inward GABA current recorded (6 ± 3% of control, n = 8; Fig. 1D). This result was unexpected, as we had predicted the block of GABA current to be largely unaffected by blue light, akin to that seen in ambient light at the start of the experiment since the sample had not been dark-adapted, so the composition of *cis*:*trans* azogabazine should be similar. On cessation of the UV or blue light exposure, the GABA currents returned to the baseline blocked state over seconds in the presence of 1 μM azogabazine and 10 μM GABA (Fig. 1D).

### 3.3 Dynamic relationship between cis and trans azogabazine

To investigate the dynamic relationship between the two azogabazine isomers, we investigated the effect of azogabazine in solution under conditions that result in three different ratios of *trans: cis* isomers. These included: a dark-adapted solution (*trans* 100: *cis* 0%); one pre-exposed for 60 s to blue light (*trans* 75: *cis* 25%); and a third exposed for the same duration to UV light (*trans* 15: *cis* 85%). NMR analysis revealed that these isomer ratios remained relatively constant for at least 12–24 h and only altered significantly over several days (Fig. 2B). All subsequent experiments were performed in near-darkness and within 3 h after the *trans*: *cis* ratios were established to ensure the stability of the isomers during experimentation. It is important to note that the immediate effects of light exposure observed in electrophysiology experiments may not be directly comparable to the data obtained from NMR analysis. The later provides assurance as to the relative time stability of the *cis* and *trans* isoforms of azogabazine.

**Fig. 3:**
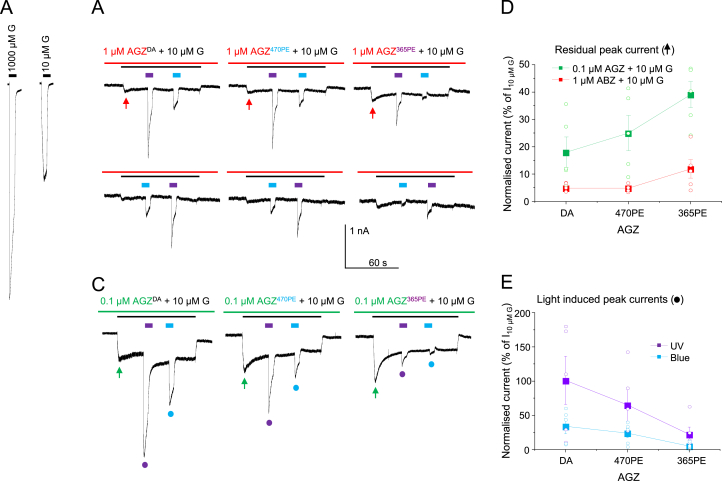
Concentration and conformational isomer dependence of azogabazine block at α1β2γ2L GABA_A_Rs. (**A**), GABA currents evoked by 10 (~EC_50_) and 1000 μM (~EC_100_) GABA (G). (**B**,**C**), In the same HEK293 cell, 1 μM (**B**) or 0.1 μM (**C**) azogabazine were pre-applied (red/green bars) before co-application (black bar) with 10 μM GABA using AGZ samples that were: dark-adapted (DA; ~100% trans); pre-exposed (PE) to 470 nm blue light: 470 PE (~75% trans); or pre-exposed to 365 nm UV light: 365 PE (~15% trans). During co-application, cells were exposed to UV (purple bar) or blue light (blue bar). In (**B**) the light protocol is applied in two sequences. Upper panel: UV then blue light. Lower panel: blue then UV light. (**D**), Peak GABA currents at the onset of co-applied GABA and azogabazine (red and green arrows in **B**,**C**) reflecting the level of inhibition caused by dark adapted, blue light exposed (470 PE), and UV light exposed (365 PE) azogabazine. Repeated measures ANOVA [0.1 μM AGZ: DA vs. 470 PE vs. 365 PE]: F(2, 8) = 29.03, p = 0.0002; followed by a Tukey multiple comparisons post hoc test: DA vs. 470 PE, p = 0.0762; DA vs. 365 PE, p = 0.0002; 470 PE vs. 365 PE, p = 0.0029; and repeated measures ANOVA [1 μM AGZ: DA vs. 470 PE vs. 365 PE]: F(2, 8) = 5.400; p = 0.0328; followed by a Tukey multiple comparisons post hoc test: DA vs. 470 PE, p = 0.9998; DA vs. 365 PE, p = 0.0492; 470 PE vs. 365 PE, p = 0.0490. (**E**), Peak GABA currents following UV or blue light exposure (5s pulses) to 0.1 μM azogabazine (DA, 470 PE or 365 PE) with 10 μM GABA. Measurements are taken from panel C indicated by the purple (UV) or blue (470 nm) dots. Repeated measures ANOVA [UV: DA vs. 470 PE vs. 365 PE]: F(2, 8) = 7.477, p = 0.0148; followed by a Tukey multiple comparisons post hoc test: DA vs. 470 PE, p = 0.2383; DA vs. 365 PE, p = 0.0118; 470 PE vs. 365 PE, p = 0.1536; and repeated measures ANOVA [Blue: DA vs. 470 PE vs. 365 PE]: F(2, 8) = 8.209; p = 0.0115; followed by a Tukey multiple comparisons post hoc test: DA vs. 470 PE, p = 0.3329; DA vs. 365 PE, p = 0.0096; 470 PE vs. 365 PE, p = 0.0854.Data points are mean ± SE of n = 5 showing individual experimental data points as open circles.

**Fig. 4:**
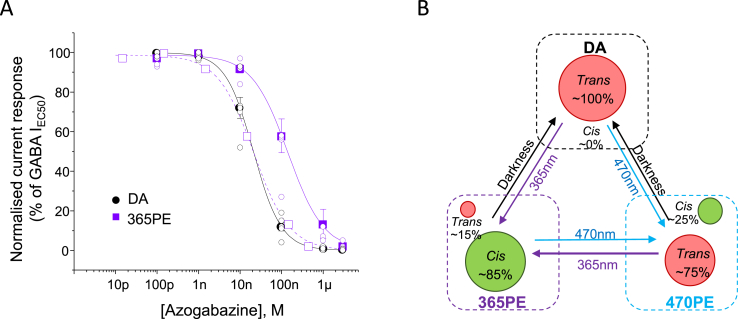
Trans isomer of azogabazine is the active inhibitory species. (**A**), Concentration-inhibition curves for dark adapted (DA; black circles) or 2 min UV pre-exposed azogabazine (365 PE; purple squares). The experiments were performed in the presence of 10 μM GABA on α1β2γ2L GABA_A_Rs. Data points are mean ± SE of n = 6. Individual data points are shown as open circles The rightward shift in the curve after UV exposure is likely to reflect the reduction in concentration of *trans*-azogabazine. Recalculating the actual *trans* isomer concentrations (15%) yields the open purple squares with fitted curve (dashed purple line) that overlaps the DA curve. (**B**), Azogabazine isomerization model. Three ratios are established between *trans:cis* isomers in: a dark-adapted sample (DA; 100% *trans-*isomer); pre-exposed to blue light sample (470 PE; 75% *trans*: 25% *cis*); and pre-exposed to UV light (365 PE; 15% *trans* and 85% *cis*). The *trans:cis* ratios of non-DA solutions are not stable, but if kept in darkness after 470 nm or 365 nm exposure, the ratio will remain constant for more than 24 h. The slow conversion to 100% *trans* takes weeks to months equating to dark adaptation.

Control membrane currents activated by 10 and 1000 μM GABA were measured in HEK293 cells expressing α1β2γ2L GABA_A_Rs (Fig. 3A). Azogabazine (0.1 or 1 μM) was first applied alone (after adaptation under different light conditions as described above) and then co-applied with 10 μM GABA to reveal an inhibited peak current (arrows, Fig. 3A–C) which slowly relaxed reflecting azogabazine attaining an equilibrium block (Fig. 3B and C). It was evident that the peak current was smaller for dark-adapted azogabazine, higher for blue light exposed azogabazine, and highest for the UV-exposed azogabazine, both at 1 μM and more evident at 0.1 μM azogabazine (Fig. 3B–D). This result is in accord with increased inhibition occurring with greater levels of *trans*-azogabazine, further suggesting that the *trans*-isomer is actively binding to, and inhibiting, GABA_A_ receptors. The extent of inhibition with both 0.1 and 1 μM azogabazine is relatively high since both these concentrations fall on the base of the concentration inhibition curve (Fig. 4A).

During the co-application of GABA and azogabazine, partially recovered GABA currents were consistently revealed during exposure to UV light and less so with blue light (Fig. 3B,C,E). To verify that the amplitudes of GABA currents evident in UV and blue light were not a consequence of the order of light exposure (e.g. and caused by receptor desensitisation), the protocol was reversed. However, exposing cells to blue light first, followed by UV light, did not alter the outcome with larger recovery currents evident in UV light (Fig. 3B, lower panel). When the experiment was repeated with 0.1 μM azogabazine residual peak GABA currents were increased compared to those exposed to 1 μM azogabazine reflecting the lower *trans*-azogabazine concentration and thus slightly reduced inhibition (Fig. 3C). This re-affirmed that *trans*-azogabazine is the active isomer blocking the GABA binding site and that the *cis* isomer is most likely unable to bind to the receptor, or at best binds with such a low affinity that this is not resolved in our experiments.

On this basis, the three GABA current levels in Fig. 3B and C represent different levels of *trans*-azogabazine bound to the GABA_A_R and thus three discernable levels of inhibition by this isomer. In a sequence of events, a pulse of UV light will effectively convert *trans*- to *cis*-azogabazine, which leads to unbinding of the azogabazine molecule from the GABA_A_R, and subsequent binding of GABA resulting in the recovery of the GABA current. However, the recovery is less than 100% of controls (in the absence of azogabazine) as not all bound *trans*-azogabazine molecules will isomerise to *cis* and unbind at the same time.

The unexpected feature of our results is why blue light elicits any current, especially when the background azogabazine solution has been pre-equilibrated in blue light. We had expected no change in the current level, or an outward current reflecting increased inhibition, but the latter was not observed (Fig. 3B). This feature probably reflects, to some extent, receptor-bound *trans*-azogabazine partly dissociating in blue light, allowing transient occupation of the orthosteric binding site by GABA thus eliciting a current. This may follow the absorption spectrum profile of *trans*-azogabazine which shows strong absorption at UV wavelengths (300–375 nm), and significant, but reduced absorption, at 450–470 nm (blue light). In blue light, the quantum yield, ɸ (= (No. azogabazine molecules photoswitching)/(No. photons absorbed)) for converting azobenzene from *trans* to *cis* (ɸ_E_) is approximately 50% that of the quantum yield for the conversion of *cis* to *trans* (ɸ_Z_) (Knoll, 2004). Hence absorption in blue light will minimally, and counterintuitively, convert some azogabazine from *trans* to *cis* isomers. If this occurs to bound *trans*-azogabazine this would lead to unbinding allowing GABA to access the orthosteric binding site and induce currents which are smaller than those observed with UV light. The sensitivity of our whole-cell recordings is sufficient to detect this small conversion of *trans* to *cis* isomer in blue light. Overall, the sequence of light exposure reveals the ability to photoregulate the extent of GABA_A_R activation by azogabazine and these results suggest that azogabazine is ideally suited for disinhibition-based studies.

To test the hypothesis that only *trans*-azogabazine binds to GABA_A_Rs to cause inhibition, azogabazine concentration-response relationships were constructed for dark-adapted and UV pre-exposed azogabazine (Fig. 4A). The inhibitory potency of dark-adapted azogabazine (~100% *trans*) was 5-fold higher than for UV pre-exposed azogabazine (pIC_50_: 7.68 ± 0.1, IC_50_: 21.1 nM; and pIC_50_: 6.98 ± 0.1, IC_50_: 104 nM, respectively). This difference in potency can be accounted for by the ratio of *trans*:*cis* azogabazine after UV exposure, since only 15% of the original concentration of active (*trans)* azogabazine will be present in the solution. By re-calculating the concentrations used to generate the inhibition curve for pre-UV exposed azogabazine to reflect that only 15% of the *trans* isomer is present, the theoretical curve overlaid the 100% *trans*-azogabazine curve for the dark-adapted solution (Fig. 4A).

To summarise, by using dark-adapted solutions (~100% *trans-*azogabazine) and establishing dynamic equilibria between *cis-* and *trans*-azogabazine, which are dominated by *trans* (~75%) after blue light and by *cis* isomers (~85%) after UV light (Fig. 4B), these data are consistent with *trans*-azogabazine predominantly binding to and inhibiting GABA_A_Rs.

### 3.4 Binding of trans-azogabazine to the GABA_A_R

**Fig. 5:**
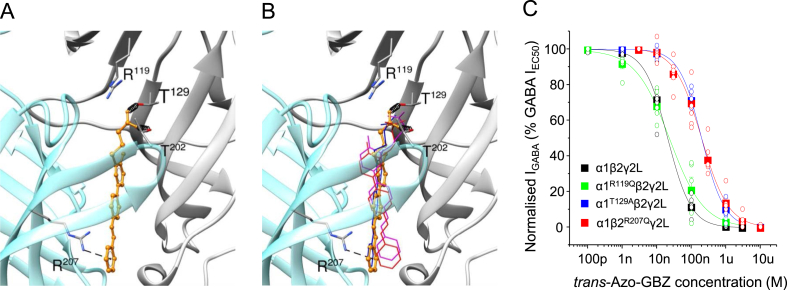
Binding site for *trans*-azogabazine at GABA_A_Rs. Predicted binding modes for *trans*-azogabazine based on the homology model of a murine α1β2γ2L GABA_A_R structure constructed using the cryo-EM structure for the human α1β3γ2L GABA_A_R (PDB ID: 6I53) as a template. (**A**), Shows the interface between β3(+) and α1(−) subunits. In the highest scoring solution (rank 1, orange) using CHEMPLP, the carboxyl tail of *trans*-azogabazine hydrogen bonds with α1^T129^ and β2^T202^ (springs) and the phenyl group engages in cation-π interactions with β2^R207^ (dashed line). (**B**), Binding modes similar to those in (**A**) were consistently predicted in the top ranked solutions (1, 3, 3) using ASP (red), GS (blue) and CS (magenta) scoring functions, respectively. The *trans*-azogabazine molecules are shown in ball and stick form (**A**), and in wire format (**B**). Interacting residues are depicted as stick models. (**C**), *Trans*-azogabazine concentration-inhibition curves for wild-type α1β2γ2L GABA_A_Rs and for the binding residue mutations: α1^T119Q^β2γ2L, α1^T129A^β2γ2L, and α1β2^R207Q^γ2L. Data points are means ± SE of n = 6–7 with individual data points shown as open circles.

Although the *trans*-isomer of azogabazine binds to GABA_A_Rs, its orientation within the orthosteric binding site at the β^+^-α^-^ subunit interface where it blocks GABA binding by virtue of its competitive inhibitory nature is unknown. To address this aspect, we used molecular docking simulations of *trans*-azogabazine into a GABA_A_R homology model for the murine α1β2γ2L isoform derived from the cryo-EM structures of the human α1β3γ2L GABA_A_R (PDB ID: 6I53; (Laverty et al., 2019; Masiulis et al., 2019). Using the GOLD docking program, we generated 50 different docking solutions based on a centroid incorporating the GABA binding site. These solutions were ranked according to the CHEMPLP (Piecewise Linear Potential) scoring function. The top solution (rank 1) exhibited preferential binding modes with the carboxyl-tail of *trans*-azogabazine forming putative hydrogen bonds with α1^T129^ and β2^T202^, and the phenyl group engaged in cation-π interactions with β2^R207^ (Fig. 5A).

In addition, we also predicted the binding modes of *trans*-azogabazine using the other three available scoring functions in GOLD: GoldScore (GS), ChemScore (CS) and Astex Statistical Potential (ASP). For each scoring function, we generated 50 different docking solutions. Binding modes similar to the orientation shown in Fig. 5A were also predicted following the top solutions ranked 1, 3, and 3 using ASP, GS and CS scoring functions, respectively (Fig. 5B).

Based on the predicted binding site residues from molecular docking, we examined the potency of *trans*-azogabazine on mutant GABA_A_Rs: α1^T129A^β2γ2L, α1β2^R207Q^γ2L and α1β2^R119Q^γ2L. The mutations, α1^T129A^ and β2^R207Q^, were made to confirm the involvement of these residues in anchoring *trans*-azogabazine in the binding site. α1^T129^ has not previously, to our knowledge, been implicated in GABA_A_R drug binding, but β2^R207^ does play such a role in binding our photoactive gabazine-benzophenone analogue, GZ-B1(Mortensen et al., 2014). In the same study, α1^T119^ was also implicated for binding GZ-B1, and we decided to include α1^T119Q^ as a negative control for azogabazine binding. The residue, β2^T202^, is known to be important for GABA binding, but as its mutation markedly reduces GABA potency, it is not used to assess *trans*-azogabazine binding (Amin and Weiss, 1993; Masiulis et al., 2019).

Both α1^T129A^β2γ2L and α1β2^R207Q^γ2L reduced azogabazine potency by 10-fold compared to wild-type receptors (pIC_50_: 6.70 ± 0.05, IC_50_: 201 nM, t(9) = 4.62, p = 0.0013, and pIC_50_: 6.73 ± 0.08, IC_50_: 187 nM, t(10) = 7.45, p < 0.0001, respectively; wt α1β2γ2L: pIC_50_: 7.68 ± 0.1, IC_50_: 21.1 nM; Fig. 4C), whereas α1β2^R119Q^γ2L did not affect azogabazine potency (pIC_50_: 7.61 ± 0.02, IC_50_: 24.4 nM, t(9) = 0.57, p = 0.5803). These results suggest that α1^T129A^β2γ2L and α1β2^R207Q^γ2L are likely important residues for binding *trans*-azogabazine in accord with the docking data. Interestingly, cryo-EM analysis of α1β3γ2L GABA_A_Rs with the competitive antagonist bicuculline bound (PBD: 6HUK; Masiulis et al., 2019) revealed that some aromatic rings of bicuculline are orientated perpendicularly to loop C in the GABA binding site. This conformational arrangement of aromatic groups is also apparent for *trans*-azogabazine in our docking simulations (Fig. 5A and B).

Overall, our data indicates that only *trans*-azogabazine can bind to GABA_A_Rs, but we decided to assess if *cis*-azogabazine could potentially bind to the orthosteric site by using computational docking studies. We searched extensively for all possible binding modes using a diverse docking protocol by sampling full ligand rotational bonds using the CHEMPLP scoring function. Out of 100 predicted docked azogabazine molecules, none of the molecular poses adopted the *cis* conformation which strongly supports the hypothesis that only *trans*-azogabazine binds to GABA_A_Rs.

### 3.5 Probing cell membrane-delimited GABA inhibition using light-activated azogabazine

To evaluate azogabazine as a tool for investigating GABA_A_R physiology in the brain, we used light to control synaptic inhibition in cultured cerebellar granule cells. As the recombinant study demonstrated that only *trans*-azogabazine can bind to the GABA binding site and inhibit the GABA_A_R, we used a dark-adapted azogabazine solution in these experiments to ensure ~100% *trans* isomer. We also used UV light to relieve azogabazine inhibition (disinhibition) by *trans-*to-*cis* isomerization. However, UV light is damaging to cells by generating free radicals, thus to ensure long-term electrophysiological recordings the intensity of the UV light was empirically optimised to maintain good cell health whilst achieving maximum photoisomerisation of azogabazine.

**Fig. 6:**
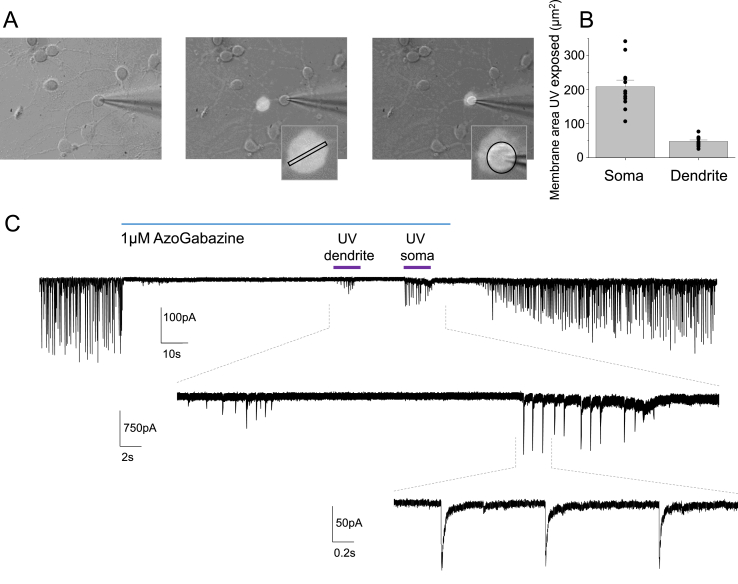
Azogabazine inhibition of IPSCs and UV illumination of somatic and dendritic membranes. (**A**), Left panel, DIC image of cultured cerebellar granule cell with whole cell recording electrode and dendritic (middle panel) and somatic (right panel) white light pilot spots. Inserts show the areas of cell membrane exposed to UV during the experiment. (**B**), Depicts the calculated areas of UV exposed cell membrane. Bars are means ± SE; individual experimental points shown as black circles; n = 12. (**C**), Top line, voltage clamp recording (at −60 mV) from a granule cell. IPSCs are blocked with 1 μM azogabazine but exhibit partial recovery during 10s UV spot exposures to dendritic or somatic membrane. (**C**), Middle line, increased resolution of IPSCs during UV exposure (dashed lines). Note higher IPSC amplitudes are observed at somatic membranes. (**C**), Lower line, IPSCs originating from the somatic membrane.

The experimental protocol was designed to regulate GABA inhibition by applying azogabazine to voltage clamped neurons and then observe the relief of inhibition by exposing membrane-delimited areas to UV light spatially-targeted to the soma or proximal dendrites. Initially, white light was used to select and set the membrane area to be targeted by UV exposure. We set the spot size and position to incorporate only the granule cell body (average soma diameter: 8.1 ± 0.4 μm; spot diameter 10.3 ± 0.6 μm). The same size spot was applied to proximal dendrites on the same cell (Fig. 6A). The area of light exposed cell membrane was smaller for the main non-branching dendrite compared to the soma, where the former, represented by a cylinder had a mean surface area of 48 ± 4.1 μm^2^ (Fig. 6A and B) and the latter, approximated by a sphere, had a mean surface area of 208 ± 20 μm^2^ (Fig. 6A and B).

**Fig. 7:**
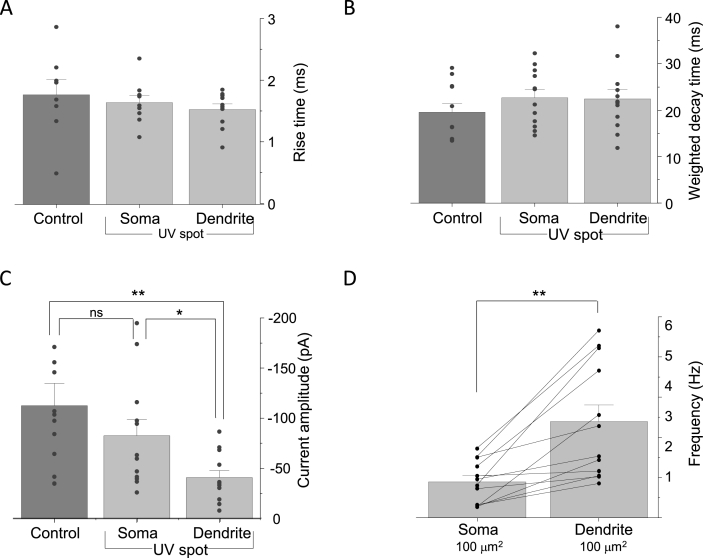
GABA inhibitory contribution from dendritic and somatic membranes. sIPSCs rise times (**A**) and weighted decay times (**B**) for synaptic inhibition at somatic and dendritic membranes exposed to 1 μM azogabazine and discerned by UV membrane delimited illumination, compared to IPSCs recorded from a control pre-azogabazine period. (**C**), Bargraph of mean IPSC amplitude from pre-gabazine control versus azogabazine treated somatic and dendritic membrane regions reversed by UV light spot. (**D**), IPSC frequencies determined for the somatic membrane and compared to the dendritic membrane when relieved of azogabazine inhibition with UV illumination. Data values are normalized to 100 μm^2^ membrane surface areas. Data are means ± SE of n = 12 experiments; individual experimental values are shown as black circles; lines between pairs are shown in **D**. *: p < 0.05, **: p < 0.01.

A range of inhibitory postsynaptic current (IPSC) amplitudes were recorded under whole-cell conditions (Fig. 6C). Applying a saturating concentration (1 μM) of azogabazine rapidly blocked all the GABA-mediated IPSCs. Subsequently, 10 s exposures to UV light during azogabazine application, directed to dendritic and subsequently somatic membranes, induced full- or part-recovery of the IPSC's (Fig. 6C) with IPSC kinetic characteristics (rise times and weighted decay times) identical to those for control IPSCs before azogabazine (Fig. 7A and B).

The IPSCs recovered during exposure to azogabazine with UV light directed to the soma were comparable in mean amplitude (−74 ± 16 pA) to that of control IPSCs (−113 ± 22 pA; t_paired_(11) = 1.45, p = 0.1681; recorded in darkness without azogabazine). By comparison, IPSC amplitudes recorded from the proximal dendritic zones showed less recovery (−41 ± 7 pA) than those recorded from the soma (t_paired_(11) = 2.73, p = 0.0195; Fig. 7C). This most likely reflects larger numbers of unblocked GABA_A_Rs at inhibitory somatic synapses compared to synapses on a single proximal dendrite.

The frequency of IPSCs will reflect the probability of transmitter release and also the number of GABA_A_R synaptic inputs (synapses) in the membrane region analysed. Hence, to compare the numbers of IPSCs at synapses from somatic and dendritic membrane areas (assuming uniform synaptic density) we normalized the area of UV exposed membrane. When normalising to a membrane area of 100 μm^2^, we observed a higher IPSC frequency from proximal dendrites compared to the soma (4.2 ± 0.9 Hz and 1.8 ± 0.3 Hz, respectively; t_paired_(11) = 3.14, p = 0.0094; Fig. 7D).

**Fig. 8:**
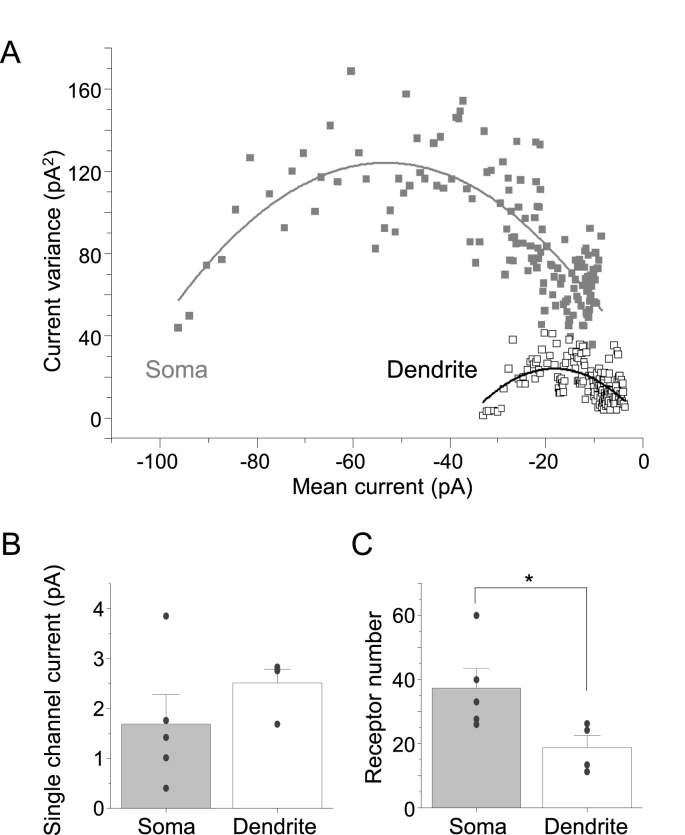
Analysing synaptic GABA_A_ receptor numbers and single channel currents. (**A**), Example peak non-stationary noise analysis for IPSCs recorded from two neurons showing current variance – mean synaptic current relationships for somatic and dendritic inhibitory synapses. Parabolic curve fits were generated as described in the Methods. (**B**,**C**), Bargraphs for synaptic GABA_A_ receptor single channel current (**B**) and mean receptor numbers open during the IPSC (**C**) populating somatic and dendritic inhibitory synapses. Bars represent means ± SE from n = 5 (soma) and 4 (dendrites), *P < 0.05, two-tailed unpaired *t*-test; individual experimental data points are shown as circles in (**B**,**C**).

To assess whether there are any differences between the numbers of synaptic GABA_A_ receptors populating somatic and dendritic inhibitory synapses we next performed non-stationary noise analysis (NSNA) of IPSCs. From the decay phases of peak-scaled IPSCs obtained from the soma and proximal dendrites (during relief of *trans*-azogabazine block with UV) we determined the single channel current and the average number of synaptic receptors open at the peak of the IPSC (Fig. 8A). We did not observe any changes to the single channel current of GABA_A_Rs resident in somatic and dendritic membranes (1.7 ± 0.59 pA and 2.5 ± 0.28, respectively; t(7) = 1.16, p = 0.2829; Fig. 8B). By contrast, the mean number of synaptic GABA_A_ receptors was greater at somatic (37.3 ± 6.2) compared with dendritic synapses (18.7 ± 3.8; t(7) = 2.40, p = 0.0474; Fig. 8C), which accords with the IPSC amplitude analysis (Fig. 7C). This difference in receptor numbers accords with an earlier observation from hippocampal pyramidal neurons where somatic synapses similarly are larger than those present on proximal dendrites (Miles et al., 1996).

**Fig. 9:**
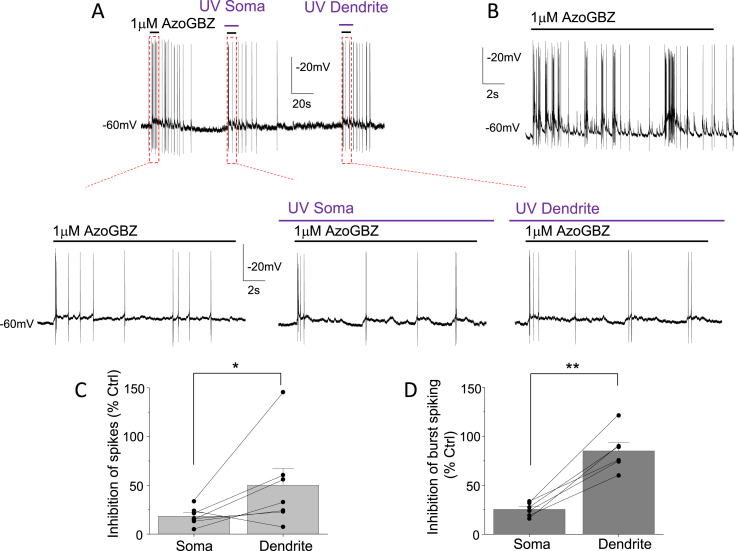
Excitability of dendritic and somatic membranes using azogabazine. (**A**), Current clamp recording (E_m_ −60 mV by constant current injection) of low frequency spiking initiated during the application of 1 μM azogabazine, and subsequently during UV light exposure of just the soma or a main dendrite. Corresponding high resolution records (red boxes) are shown in the left, middle and right panels below. (**B**), Example cell (at −60 mV) displaying higher spike frequency in current clamp in the presence of 1 μM azogabazine, including spike bursting. (**C**), Bargraph for the level of inhibition of individual spike firing by azogabazine after isomerization with UV light applied to the somatic and dendritic membranes, n = 12 cells. (**D**), Bargraph for inhibition of burst firing of spikes from somatic and dendritic membrane by reducing azogabazine block with UV light, n = 4 cells. Data are means ± SE; individual experimental paired data points are shown as black circles connected with lines. *: p < 0.05, **: p < 0.01.

To assess neuronal excitability and GABA inhibition, current clamp recordings were performed from cultured granule cells to monitor action potential generation in the presence of 1 μM azogabazine before and after applying UV light. In the presence of azogabazine (control, before UV) the spike frequency was 8.4 ± 3.3 Hz, with the majority of cells presenting individual APs at lower frequencies (8 out of 12 cells; Fig. 4, Fig. 9 cells displaying burst firing (Fig. 9B). In burst firing cells, the burst frequency was 1.1 ± 0.3 Hz where each burst contained on average 11 ± 3 individual spikes.

We used membrane-delimited UV light exposures to the soma and proximal dendrites on each cell, photoisomerizing azogabazine from *trans* to *cis*, leading to off-binding of the antagonist and thereby increased activation of GABA_A_Rs, which ultimately resulted in reduced spike firing. Normalisation to the area of the UV exposed membrane, revealed that the generation of individual spikes were inhibited significantly more in the proximal dendritic zones than in the somatic membrane (50 ± 17% versus 18 ± 3%, t_paired_(6) = 2.53, p = 0.0448; Fig. 9C) and a similar observation was made for spike burst generation (60 ± 9% compared to 25 ± 3%, t_paired_(5) = 5.90, p = 0.0097; Fig. 9D).

These results accord with the observations of IPSC frequency in voltage clamp and suggest that GABA_A_Rs on proximal dendritic membranes have a dominant inhibitory role in determining the input - output relationship in cerebellar granule cells, highlighting the usefulness of azogabazine as a tool for studying membrane-delimited specific GABA inhibition.

## 4 Discussion

The photochemical tool box for GABA_A_Rs has been expanded by studies specifically designed to create new photoprobes for investigating synaptic inhibition (Lin et al., 2015; Yue et al., 2012; Mortensen et al., 2014). However, except for the antagonists GZ-B1(Mortensen et al., 2014) and azogabazine, other photoprobes have been based on a GABA agonist or on positive allosteric modulators of the GABA_A_R (Mortensen et al., 2014; Lin et al., 2015; Mortensen and Smart, 2015; Yue et al., 2012). Counterintuitively, the photo-agonist (Lin et al., 2014) and positive allosteric modulators (Yue et al., 2012) all function as inhibitors, and although it is useful to have a photo-chemical reagent capable of limiting inhibition, we wanted to start with a recognised competitive inhibitor as the basis for our new photo-switchable GABA_A_R probe.

The adoption of light-based methods to regulate neural cellular and circuit activity is increasingly advantageous offering non-invasive reversible control of GABA inhibition. It is also possible to control circuit activity using caged compounds (Ellis-Davies, 2007; Trigo et al., 2009), optogenetics based on light-regulated membrane proteins such as channelrhodopsins and halorhodopsins (Yizhar et al., 2011; Gradinaru et al., 2010), and chemogenetics (Sternson and Roth, 2014). Furthermore, the expression of mutated neurotransmitter proteins in neurons that enable sensitivity to light- and chemically-activated probes have also gained increasing traction (Kramer et al., 2013; Lin et al., 2014; Mortensen and Smart, 2015). Whilst many of these techniques will necessarily rely on the expression of recombinant proteins in neurons, it is also important to harness optical techniques for manipulating the activity of *native* endogenous proteins without the need to express receptor variants. This requires the development of photochemical ligands that can bind to native receptors or ion channels and respond to different wavelengths of light to modulate the activity of neurons.

The unique feature of the azobenzene group, and related derivatives, is the ability to photoisomerise between *cis*- and *trans*-isomers, where the *cis*-isomer is less stable and will thermally relax to the more stable *trans*-state over time under zero light (dark adaptation) conditions. They have other significant advantages exemplified by a high quantum yield, a high degree of photostability, and most important, they offer an easily reversible photosensitive tool (Beharry and Woolley, 2011) for investigating receptor function. These properties have been exploited for studying G-protein coupled receptors (Spangler and Bruchas, 2017), voltage-gated ion channels (Szobota and Isacoff, 2010; Banghart et al., 2004; Kramer et al., 2013) and ligand-gated ion channels (Browne et al., 2014; Klippenstein et al., 2017).

Azobenzene analogues have been tethered directly to voltage-gated potassium channels to regulate ion flux through the channel depending on the conformational status of the azobenzene group (Banghart et al., 2004; Szobota and Isacoff, 2010). Agonists like GABA or glutamate have also been chemically attached to azobenzene and tethered via maleimides to cysteines previously introduced into GABA_A_Rs or NMDA receptors near neurotransmitter binding sites, functioning as switches, entering or exiting the binding site upon light stimulation (Lin et al., 2014; Berlin et al., 2016; Levitz et al., 2013, 2016). These examples require genetic manipulation of the protein of interest, but some studies have also successfully utilised the photoswitch ability of azobenzene chemically linked to ligands that bind to native receptors (Yue et al., 2012; Volgraf et al., 2007), and azogabazine belongs to this latter class of photoactivatable ligands.

The isomerization of azobenzene is controlled by exposure to UV and blue light, the former displacing the photostationary state in favour of the *cis* isomer and the latter in favour of the *trans* isomer. Furthermore, in complete darkness, a thermal mechanism operates shifting the equilibrium towards the thermodynamically-stable *trans-*azogabazine (~100%). It is important to note that the ratios between *cis*- and *trans*-isomers can be altered following prolonged exposure to light, and that these ratios are relatively stable for many hours if kept in darkness. Eventually after many days in darkness, the *trans*-isomer will become completely dominant.

From our recombinant experiments, we deduced that the *trans*-isomer preferentially binds to the GABA binding site, and thus it was only the concentration of the *trans*-isomer that was relevant for blocking the GABA_A_R. Accordingly, the more *trans*-azogabazine present, the greater the block of GABA_A_Rs. This accounts for why dark-adapted azogabazine solutions (100% *trans*-azogabazine) were more potent at inhibiting GABA_A_Rs compared to blue light pre-exposed solutions (75% *trans*-azogabazine), and especially the UV light pre-exposed solutions (15% *trans*-azogabazine), with their lower levels of the *trans* isomer.

The potential modes by which *trans*-azogabazine could bind at the GABA binding site with the azobenzene group pointing towards the cell membrane, and the likely direct involvement of especially α1^T129^ and β2^R207^ was resolved from computational docking to the murine α1β2γ2L receptor. This revealed α1^T129^, which has previously been implicated in the binding of GABA and the agonist muscimol (Kloda and Czajkowski, 2007; Bergmann et al., 2013), and also β2^R207^ implicated in GZ-B1 binding (Mortensen et al., 2014). Mutagenesis of both T129 (α1^T129A^) and R207 (β2^R207Q^) further suggested key roles for these residues in azogabazine binding.

UV light was used to relieve azogabazine-inhibition in delimited cell membrane domains of single neurons and to investigate the relative properties of synaptic inhibition and consequences for neuronal excitability. This enabled the contribution of synaptic inputs to the somatic and proximal dendritic membranes to be segregated for cerebellar granule cells. These experiments revealed that GABA-induced IPSC amplitudes were higher at the soma compared to the dendrites, which is indicative of larger synapses containing higher numbers of GABA_A_Rs assuming similar levels of GABA release (Miles et al., 1996). Furthermore, the frequency of IPSCs was higher on dendrites compared with the soma, when normalized to the membrane area, which suggests a higher number of GABAergic synapses in the proximal dendritic zone per area unit when compared to the soma. It is possible that cerebellar granule neurons in primary cultures may display different distributions of GABA_A_Rs on the cell surface compared with those found *in situ*. However, it seems unlikely given the differential distribution of neurotransmitter receptors between soma, axon initial segment and dendrites reported in hippocampal pyramidal neurons (Miles et al., 1996). Furthermore, such an unequal distribution is also evident from photo-uncaging experiments for GABA_A_Rs (Kanemoto et al., 2011; Matsuzaki et al., 2010), although these studies did not discriminate between synaptic and extrasynaptic receptors.

The kinetic parameters of sIPSCs (rise times and weighted decay times) were identical in our sampling of somatic and dendritic inhibition presumably reflecting the presence of similar populations of synaptic GABA_A_R subtypes in these membrane delimited domains. Indeed, for cerebellar granule cells, the same types of synaptic GABA_A_Rs (α1β2/3γ2, α6β2/3γ2, α1α6β2/3γ2) have been reported to be present both in the soma and along the dendrites, along with extrasynaptic α6β2/3δ GABA_A_Rs (Nusser et al., 1998). The observed differences in size and number of GABAergic synapses between somatic and proximal dendritic regions had a clear impact on spike frequency where integrative GABA_A_R inhibition was clearly more prominent in dendritic membranes.

In conclusion, we have characterised a new photoisomerisable tool, azogabazine, where only the *trans*-isomer potently blocks GABA_A_Rs and can readily and substantially dissociate from the receptor as a consequence of forming the *cis* isomer after exposure to direct UV light. Azogabazine enabled the non-uniformity of GABA_A_R synapses in terms of average receptor numbers in inhibitory synapses (IPSC current amplitude) and numbers of synapses (IPSC frequency), between somatic and proximal dendritic regions to be interrogated. By adopting this new optopharmacological approach using an photosensitive reversible GABA antagonist, we conclude that proximal dendrites on cerebellar granule cells carry greater inhibitory ‘weight’ compared to their somatic counterparts in controlling neuronal spike output.

## Funding and acknowledgements

This study was funded by the 10.13039/501100000265Medical Research Council (MRC) (TGS, MM), 10.13039/501100000268Biotechnology and Biological Sciences Research Council (BBSRC) (JRB), a studentship from Pfizer (RH). APP thanks the MRC Laboratory for Molecular Biology and the BBSRC (lab. of Prof Julian Gough) for support and David Houldershaw, Birkbeck, for computer support.

## CRediT authorship contribution statement

**Martin Mortensen:** Data curation, Formal analysis, Writing - original draft, Writing - review & editing. **Rosemary Huckvale:** Writing - review & editing. **Arun P. Pandurangan:** Formal analysis, Data curation. **James R. Baker:** Conceptualization, Resources, Writing - original draft, Supervision. **Trevor G. Smart:** Conceptualization, Funding acquisition, Resources, Supervision, Writing - original draft, Writing - review & editing.

## References

[bib1] Amin J., Weiss D.S. (1993). GABA_A_ receptor needs two homologous domains of the β-subunit for activation by GABA but not by pentobarbital. Nature.

[bib2] Banghart M., Borges K., Isacoff E., Trauner D., Kramer R.H. (2004). Light-activated ion channels for remote control of neuronal firing. Nat. Neurosci..

[bib3] Beharry A.A., Woolley G.A. (2011). Azobenzene photoswitches for biomolecules. Chem. Soc. Rev..

[bib4] Bergmann R., Kongsbak K., Sørensen P.L., Sander T., Balle T. (2013). A unified model of the GABA_A_ receptor comprising agonist and benzodiazepine binding sites. PloS One.

[bib5] Berlin S., Szobota S., Reiner A., Carroll E.C., Kienzler M.A., Guyon A., Xiao T., Trauner D., Isacoff E.Y. (2016). A family of photoswitchable NMDA receptors. elife.

[bib6] Bogdanov Y., Michels G., Armstrong-Gold C., Haydon P.G., Lindstrom J., Pangalos M., Moss S.J. (2006). Synaptic GABA_A_ receptors are directly recruited from their extrasynaptic counterparts. EMBO J..

[bib7] Brickley S.G., Cull-Candy S.G., Farrant M. (1999). Single-channel properties of synaptic and extrasynaptic GABA_A_ receptors suggest differential targeting of receptor subtypes. J. Neurosci..

[bib8] Brickley S.G., Mody I. (2012). Extrasynaptic GABA_A_ receptors: their function in the CNS and implications for disease. Neuron.

[bib9] Broichhagen J., Trauner D. (2014). The *in vivo* chemistry of photoswitched tethered ligands. Curr. Opin. Chem. Biol..

[bib10] Browne L.E., Nunes J.P., Sim J.A., Chudasama V., Bragg L., Caddick S., Alan N.R. (2014). Optical control of trimeric P2X receptors and acid-sensing ion channels. Proc. Natl. Acad. Sci. U.S.A..

[bib11] Ellis-Davies G.C.R. (2007). Caged compounds: photorelease technology for control of cellular chemistry and physiology. Nat. Methods.

[bib12] Glykys J., Mann E.O., Mody I. (2008). Which GABA_A_ receptor subunits are necessary for tonic inhibition in the hippocampus?. J. Neurosci..

[bib13] Gradinaru V., Zhang F., Ramakrishnan C., Mattis J., Prakash R., Diester I., Goshen I., Thompson K.R., Deisseroth K. (2010). Molecular and cellular approaches for diversifying and extending optogenetics. Cell.

[bib14] Hines R.M., Davies P.A., Moss S.J., Maguire J. (2012). Functional regulation of GABA_A_ receptors in nervous system pathologies. Curr. Opin. Neurobiol..

[bib15] Huckvale R., Mortensen M., Pryde D., Smart T.G., Baker J.R. (2016). Azogabazine; a photochromic antagonist of the GABA_A_ receptor. Org. Biomol. Chem..

[bib16] Iqbal F., Ellwood R., Mortensen M., Smart T.G., Baker J.R. (2011). Synthesis and evaluation of highly potent GABA_A_ receptor antagonists based on gabazine (SR-95531). Bioorg. Med. Chem. Lett.

[bib17] Jacob T.C., Moss S.J., Jurd R. (2008). GABA_A_ receptor trafficking and its role in the dynamic modulation of neuronal inhibition. Nat. Rev. Neurosci..

[bib18] Jones G., Willett P., Glen R.C., Leach A.R., Taylor R. (1997). Development and validation of a genetic algorithm for flexible docking. J. Mol. Biol..

[bib19] Kanemoto Y., Matsuzaki M., Morita S., Hayama T., Noguchi J., Senda N., Momotake A., Arai T., Kasai H. (2011). Spatial distributions of GABA receptors and local inhibition of Ca^2+^ transients studied with GABA uncaging in the dendrites of CA1 pyramidal neurons. PloS One.

[bib20] Kang J.Q., Macdonald R.L. (2009). Making sense of nonsense GABA_A_ receptor mutations associated with genetic epilepsies. Trends Mol. Med..

[bib21] Klippenstein V., Hoppmann C., Ye S., Wang L., Paoletti P. (2017). Optocontrol of glutamate receptor activity by single side-chain photoisomerization. eLife.

[bib22] Kloda J.H., Czajkowski C. (2007). Agonist-, antagonist-, and benzodiazepine-induced structural changes in the α1Met^113^-Leu^132^ region of the GABA_A_ receptor. Mol. Pharmacol..

[bib23] Knoll H., Horspool W., Lenci F. (2004). Photoisomerism of azobenzenes.

[bib24] Korpi E.R., Gründer G., Lüddens H. (2002). Drug interactions at GABA_A_ receptors. Prog. Neurobiol..

[bib25] Kramer R.H., Mourot A., Adesnik H. (2013). Optogenetic pharmacology for control of native neuronal signaling proteins. Nat. Neurosci..

[bib26] Krivov G.G., Shapovalov M.V., Dunbrack R.L. (2009). Improved prediction of protein side-chain conformations with SCWRL4. Proteins.

[bib27] Laverty D., Desai R., Uchariski T., Masiulis S., Stec W.J., Malinauskas T., Zivanov J., Pardon E., Steyaert J., Miller K.W., Aricescu A.R. (2019). Cryo-EM structure of the human α1β3γ2 GABA_A_ receptor in a lipid bilayer. Nature.

[bib28] Levitz J., Pantoja C., Gaub B., Janovjak H., Reiner A., Hoagland A., Schoppik D., Kane B., Stawski P., Schier A.F., Trauner D., Isacoff E.Y. (2013). Optical control of metabotropic glutamate receptors. Nat. Neurosci..

[bib29] Levitz J., Popescu A.T., Reiner A., Isacoff E.Y. (2016). A toolkit for orthogonal and in vivo optical manipulation of ionotropic glutamate receptors. Front. Mol. Neurosci..

[bib30] Lin W.C., Davenport C.M., Mourot A., Vytla D., Smith C.M., Medeiros K.A., Chambers J.J., Kramer R.H. (2014). Engineering a light-regulated GABA_A_ receptor for optical control of neural inhibition. ACS Chem. Biol..

[bib31] Lin W.C., Tsai M.C., Davenport C., Smith C., Veit J., Wilson N., Adesnik H., Kramer R. (2015). A comprehensive optogenetic pharmacology toolkit for in-vivo control of GABA_A_ receptors and synaptic inhibition. Neuron.

[bib32] Luscher B., Fuchs T., Kilpatrick C. (2011). GABA_A_ receptor trafficking-mediated plasticity of inhibitory synapses. Neuron.

[bib33] Masiulis S., Desai R., Uchariski T., Serna Martin I., Laverty D., Karia D., Malinauskas T., Zivanov J., Pardon E., Kotecha A., Steyaert J., Miller K.W., Aricescu A.R. (2019). GABA_A_ receptor signalling mechanisms revealed by structural pharmacology. Nature.

[bib34] Matsuzaki M., Hayama T., Kasai H., Ellis-Davies G.C.R. (2010). Two-photon uncaging of γ-aminobutyric acid in intact brain tissue. Nat. Chem. Biol..

[bib35] Miles R., Toth K., Gulyas A.I., Hajos N., Freund T.F. (1996). Differences between somatic and dendritic inhibition in the hippocampus. Neuron.

[bib36] Möhler H. (2012). The GABA system in anxiety and depression and its therapeutic potential. Neuropharmacology.

[bib37] Mortensen M., Iqbal F., Pandurangan A.P., Hannan S., Huckvale R., Topf M., Baker J.R., Smart T.G. (2014). Photo-antagonism of the GABA_A_ receptor. Nat. Commun..

[bib38] Mortensen M., Smart T.G. (2015). Neuronal inhibition under the spotlight. Neuron.

[bib39] Mortensen M., Smart T.G. (2006). Extrasynaptic αβ subunit GABA_A_ receptors on rat hippocampal pyramidal neurons. J. Physiol. (Lond.).

[bib40] Mortensen M., Smart T.G. (2007). Single-channel recording of ligand-gated ion channels. Nat. Protoc..

[bib41] Nusser Z., Sieghart W., Somogyi P. (1998). Segregation of different GABA_A_ receptors to synaptic and extrasynaptic membranes of cerebellar granule cells. J. Neurosci..

[bib42] Pettersen E.F., Goddard T.D., Huang C.C., Couch G.S., Greenblatt D.M., Meng E.C., Ferrin T.E. (2004). UCSF Chimera - a visualization system for exploratory research and analysis. J. Comput. Chem..

[bib43] Ramamoorthi K., Lin Y. (2011). The contribution of GABAergic dysfunction to neurodevelopmental disorders. Trends Mol. Med..

[bib44] Rudolph U., Möhler H. (2014). GABA_A_ receptor subtypes: therapeutic potential in Down syndrome, affective disorders, schizophrenia, and autism. Annu. Rev. Pharmacol. Toxicol..

[bib45] Sali A., Blundell T.L. (1993). Comparative protein modelling by satisfaction of spatial restraints. J. Mol. Biol..

[bib46] Sieghart W., Sperk G. (2002). Subunit composition, distribution and function of GABA_A_ receptor subtypes. Curr. Top. Med. Chem..

[bib47] Sieghart W., Savić M.M. (2018). International Union of Basic and Clinical Pharmacology. CVI: GABA_A_ receptor subtype- and function-selective ligands: key issues in translation to humans. Pharmacol. Rev..

[bib48] Smart T.G., Paoletti P., Sheng M., Sabatini B.L., Sudhof T.C. (2012). Synaptic neurotransmitter-gated receptors. The Synapse.

[bib49] Spangler S.M., Bruchas M.R. (2017). Optogenetic approaches for dissecting neuromodulation and GPCR signaling in neural circuits. Curr. Opin. Pharmacol..

[bib50] Sternson S.M., Roth B.L. (2014). Chemogenetic tools to interrogate brain functions. Annu. Rev. Neurosci..

[bib51] Szobota S., Isacoff E.Y. (2010). Optical control of neuronal activity. Annu. Rev. Biophys..

[bib52] Thomas P., Mortensen M., Hosie A.M., Smart T.G. (2005). Dynamic mobility of functional GABA_A_ receptors at inhibitory synapses. Nat. Neurosci..

[bib53] Trigo F.F., Papageorgiou G., Corrie J.E.T., Ogden D. (2009). Laser photolysis of DPNI-GABA, a tool for investigating the properties and distribution of GABA receptors and for silencing neurons in situ. J. Neurosci. Methods.

[bib54] Volgraf M., Gorostiza P., Szobota S., Helix M.R., Isacoff E.Y., Trauner D. (2007). Reversibly caged glutamate: a photochromic agonist of ionotropic glutamate receptors. J. Am. Chem. Soc..

[bib55] Yamasaki T., Hoyos-Ramirez E., Martenson J.S., Morimoto-Tomita M., Tomita S. (2017). GARLH family proteins stabilize GABA_A_ receptors at synapses. Neuron.

[bib56] Yizhar O., Fenno L., Davidson T., Mogri M., Deisseroth K. (2011). Optogenetics in neural systems. Neuron.

[bib57] Yue L., Pawlowski M., Dellal S.S., Xie A., Feng F., Otis T.S., Bruzik K.S., Qian H., Pepperberg D.R. (2012). Robust photoregulation of GABA_A_ receptors by allosteric modulation with a propofol analogue. Nat. Commun..

[bib58] Zhu M., Zhou H. (2018). Azobenzene-based small molecular photoswitches for protein modulation. Org. Biomol. Chem..

